# In vivo and in vitro safety evaluation of fermented *Citrus sunki* peel extract: acute and 90-day repeated oral toxicity studies with genotoxicity assessment

**DOI:** 10.1186/s12906-020-03079-z

**Published:** 2020-10-06

**Authors:** Jin-Sung Park, Eun-Young Cho, Yun-Soon Kim, Euna Kwon, Kang-Min Han, Seung-Yup Ku, Chul-Woo Jung, Jun-Won Yun, Jeong-Hwan Che, Byeong-Cheol Kang

**Affiliations:** 1 Department of Experimental Animal Research, Biomedical Research Institute, Seoul National University Hospital, Seoul, Republic of Korea; 2 Graduate School of Translational Medicine, Seoul National University College of Medicine, 101 Daehak-ro, Jongno-gu, Seoul, 03080 Republic of Korea; 3 Department of Pathology, Dongguk University Ilsan Hospital, Goyang, South Korea; 4 Department of Obstetrics and Gynecology, Seoul National University Hospital, Seoul, Republic of Korea; 5 Department of Anesthesiology and Pain Medicine, Seoul National University Hospital, Seoul, Republic of Korea; 6 Department of Biotechnology, The Catholic University of Korea, Bucheon, Republic of Korea; 7 Biomedical Center for Animal Resource and Development, Seoul National University College of Medicine, Seoul, Republic of Korea; 8 Designed Animal and Transplantation Research Institute, Institute of GreenBio Science Technology, Seoul National University, Pyeongchang-gun, Gangwon-do Republic of Korea

**Keywords:** *Citrus sunki*, Fermented peel extract, Acute toxicity, Subchronic toxicity, Mutagenicity, Clastogenicity

## Abstract

**Background:**

*Citrus sunki* Hort. ex Tanaka peel has been traditionally used as an ingredient in folk medicine due to its therapeutic effects on promotion of splenic health and diuresis as well as relief of gastrointestinal symptoms. Although a growing interest in health-promoting natural products and the development of highly concentrated products have facilitated consumption of *C. sunki* peel, its safety assessment has not been explored, posing a potential health risk. In this study, we carried out a series of systemic and genetic toxicity tests on fermented *C. sunki* peel extract (FCPE) to provide the essential information required for safe use in human.

**Methods:**

We conducted acute and 90-day repeated oral toxicity studies in Sprague-Dawley rats to evaluate systemic toxicity, and three genotoxicity assays to measure bacterial mutation reversion, cellular chromosome aberration and in vivo micronucleus formation.

**Results:**

Single oral administration of FCPE did not cause any clinical signs and lethality in all animals, establishing LD50 to be over 2000 mg/kg BW. Repeated administration of up to 2000 mg/kg BW FCPE for 90 days revealed no test substance-related toxicity as demonstrated in analysis of body weight gain, food/water intake, blood, serum biochemistry, organ weight and histopathology, collectively determining that the no-observable-adverse-effect-level of FCPE is over 2000 mg/kg BW. In addition, we detected no mutagenicity and clastogenicity in FCPE at 5000 μg/plate for the in vitro assays and 2000 mg/kg BW for the in vivo micronucleus test.

**Conclusion:**

FCPE did not cause systemic and genetic toxicity in our model systems at the tested dose levels. These results suggest a guideline for safe consumption of *C. sunki* peel in human.

## Background

Citrus species have been widely consumed since ancient times. It belongs to a large family of Rutaceae which includes approximately 160 genera and more than 1600 species. Globally, citrus species, especially *C. sinensis* (sweet oranges) and *C. reticulata* (tangerines) as the most popular citrus fruits, are commercially cultivated in many countries, producing over 124 million tons in 2016 [1]. Beside their popularity as fruit, citrus species have been traditionally used as medicinal ingredients due to their pharmacological effects [2].

Citrus species has been known as a rich source of various biologically active compounds; a recent review reported that *C. sinensis* contains approximately 150 chemical compounds including flavonoids, steroids, hydroxyamides, alkanes and fatty acids, coumarins, peptides, carbohydrates, carbamates and alkylamines, carotenoids and volatile compounds [3]. Among these, citrus species are known as a rich source of various flavonoids [4], which has been a target of growing interests due to their therapeutic efficacy on chronic debilitating diseases such as cancer, cardiovascular diseases, neurodegenerative diseases and diabetes through interaction with key enzymes or modulation of gene expression as also reviewed in [5]. Flavonoids identified in citrus are mainly categorized into 1) flavone glycosides such as hesperidin and naringin, and 2) polymethoxy flavonoids including nobiletin and tangeretin. Interestingly, these compounds are more abundantly contained in the peel than other parts of citrus [6, 7], supporting the preferable use of citrus peels in folk medicine.

*C. sunki* Hort. ex Tanaka, locally called as ‘*Jinkyool*’ or ‘*Sankyool*’, is one of the oldest native citrus species in Jeju island, South Korea, as the first record appeared in the eleventh century [8]. Although *C. sunki* has been an unfavorable crop for commercial cultivation and mass production due to a low economical value (i.e., small size and poor taste), its dried peel ‘*Jinpi*’ has been preferably used as a herbal medicine ingredient for its therapeutic effects described in a Korean medical book Donguibogam published in 1613. Recently, several studies experimentally confirmed its pharmacological efficacy in various diseases including obesity [9, 10], inflammation and allergy [11, 12], and neuronal damage [13, 14]. Moreover, the levels of flavonoids in *C. sunki* peel has been found to be high among citrus species [15], leading to increase of its utilization as herbal drugs and supplements. Fermented *C. sunki* peel extract (FCPE) has been recently developed by refining the production process to further increase the concentration of pharmacologically active components such as flavonoids [16].

Citrus peel has been empirically regarded as relatively safe for human consumption due to a long history of use with low occurrence of side effects and therefore most of them have not been a subject of toxicity studies. However, low levels of toxicity associated with high doses of citrus peel-derived flavonoids [17, 18] and the increased risk of overdosing and misuse along with mass production and utilization of highly concentrated products necessitated an urgent attention to assessment of their potential health risk. To date, toxicity information on *C. sunki* peel extract, unlike the extensive investigation into its pharmacological effectiveness [9–14, 19], has not been available due to lack of investigation. In this study, we therefore conducted a systematic toxicity study on FCPE using oral acute and 90-day repeated toxicity tests with evaluation of genotoxic potential following the OECD guidelines as part of a preclinical toxicity study for its regulatory assessment as functional food. Our results show the safe range of FCPE in in vivo and in vitro model systems, providing an essential guideline for safe use of *C. sunki* peel extract in human.

## Methods

### Preparation of fermented *C. sunki* Hort. ex Tanaka peel extract

FCPE was prepared by EM Life Co. (Jeju, Korea) as described elsewhere [16]. Briefly, the peel from *C. sunki* harvested in Jeju Island, Korea, was purchased and authenticated by EM Life Co. Voucher specimen (SNUH 10005) has been deposited at Department of Experimental Animal Research, Biomedical Research Institute, Seoul National University Hospital. Before fermentation, the peel was dried at 40 °C for a week and cut into 1 ~ 2 cm pieces. Fermentation was carried out in the sterilized filtrate of broth cultured with 5% (w/w) *Lactobacillus plantarum* (ATCC 8014, 3 × 10^6^/mL) and 5% (w/w) *Saccharomyces cerevisiae* (IFO 0203, 4 × 10^6^/mL) at 38 °C for 1 week. Then, the fermented peel underwent extraction at 98 ~ 100 °C for 2 h after mixing with 20 times volume of water. The extract was then filtered, converted into powder using a freeze dryer and stored with light protection at − 20 °C. The quality of the extract was determined by analyzing the levels of *p*-synephrine, nobiletin and tangeretin as reference compounds using HPLC (Waters 2995, USA).

### Animals

SPF Sprague-Dawley (SD) rats were chosen for this study as suggested in OECD guidelines due to abundance of historical and toxicity data. 5-Week old animals were purchased from Orient Bio Inc. (Gyeonggi-do, Korea) and only healthy animals were used in the study after two-week acclimation. As previously described [20], animals were housed in polycarbonate cages containing aspen bedding materials in an environment-controlled animal room at the AAALAC International-accredited animal facility (#001160) of Seoul National University Hospital. Animals were provided with sterilized laboratory rodent diet (LabDiet 5002, PMI Nutrition International) and autoclaved water *at libitum*. All experimental procedures were carried out during the light cycle in a separate laboratory. Prior to the start of toxicity tests, animals were weighed and randomly assigned to each group based on their body weight according to the stratified continuous randomization method. All experiments were approved by the Institutional Animal Care and Use Committee in Seoul National University Hospital in accordance with Guide for the Care and Use of Laboratory Animals (8th edition) [21]. Our toxicity studies were performed in compliance with the relevant OECD test guidelines [22–26] and Good Laboratory Practice regulations published by Korea Food Drug Administration [27].

### Oral toxicity study

Acute toxicity test was carried out as a limit test using 5 animals/gender/group (*n* = 20, male; 273.6 ~ 301.2 g and female; 174.5 ~ 207.5 g at the initiation of administration) with a single gavage of either 0 or 2000 mg FCPE homogeneously suspended in 10 mL vehicle (ddH_2_O)/kg BW followed by monitoring of clinical signs and body weight changes for 14 days. At the end of the observation period, all animals were sacrificed and examined for macroscopic legions on major organs.

For the 90-day repeated oral toxicity test, total 80 animals (*n* = 10 animals/gender/group, male; 248.0 ~ 275.4 g and female; 174.4 ~ 201.2 g at the initiation of administration) were orally administered with a dose of 0, 500, 1000 and 2000 mg/kg BW of FCPE daily for 90 days. During the administration period, animals were monitored daily for clinical signs with weekly measurement of body weight and feed/water consumption. Ophthalmological examination was performed on 5 animals/gender/group. At the end of the study, all surviving animals were euthanized by exsanguination via the vena cava after deep anesthesia with isoflurane and necropsy was performed.

### Analysis of urine, blood and serum biochemistry

In the last week of 90-day toxicity study, urine was collected from 5 animals/gender/group and analyzed using a urinalysis stick (Roche) for pH, leukocyte, nitrite, ketone body, urobilinogen, bilirubin, glucose, occult blood. Specific gravity was measured by refractometry.

Whole blood was collected at necropsy from the vena cava and used for hematological and serum biochemical analysis. Whole blood collected in an EDTA tube was used for measurement of total and differential cell counts, hemoglobin, hematocrit, platelet and RBC indices using an MS9–5 Animal Blood Counter (Melet schloesing laboratoires), and reticulocytes using a Sysmex XE-2100 automated Blood Cell Counter (Sysmex). Blood coagulation capacity was assessed by measuring partial thromboplastin time and activated partial thromboplastin time in a Coagulation Analyzer (STA-R Evolution, Diagnostica Stago).

Serum was separated by brief centrifugation of coagulated whole blood and analyzed for blood urea nitrogen, total cholesterol, total protein, albumin, total bilirubin, alkaline phosphatase, aspartate aminotransferase, alanine aminotransferase, creatinine, triglycerides, glucose, potassium, chlorine, calcium, inorganic phosphorus and sodium using a Hitachi 7070 automatic chemistry analyzer (Hitachi).

### Necropsy and histopathology

All major organs were excised immediately after gross examination and weighed. Then, testes and epididymides were fixed in Bouin’s solution, eyes and harderian glands in Davidson solution, and all other organs in 10% neutral buffered formalin. Femora and nasal cavities were decalcified after fixation. Sufficiently fixed organs were embedded in paraffin after dehydration in graded ethanol and xylene, and stained with hematoxylin and eosin after sectioning into 2–3 μm slices. Histopathological evaluation was carried out by a pathology specialist.

### Genotoxicity assays

All the genotoxicity studies on FCPE here were carried out as previously described [28]. Briefly, the reverse mutation test was performed using five tester strains including *Salmonella typhimurium* TA98, TA100, TA1535, TA1537, and *Escherichia coli* WP2(*uvrA*) (MOLTOX Molecular Toxicology, USA). After determining cytotoxicity, all strains were treated in triplicate with either vehicle or a dose of FCPE (312.5, 625, 1250, 2500 and 5000 μg/plate) or appropriate positive controls. For metabolic activation, S9 mix (Orient Yeast, Japan) was co-incubated with FCPE at 37 °C for 30 min. After treatment, the tester strains were grown on either histidine or tryptophan-deficient agar plates at 37 °C for 48 h and the colonies formed were counted.

Chromosome aberration test was performed on Chinese Hamster Lung (CHL) cells. Cells plated at 1 × 10^5^ were grown overnight and treated in duplicate with either non-toxic doses of FCPE (1250, 2500 and 5000 μg/mL) or controls for 6 h or 24 h. For treatment for 6 h, we employed an additional group for metabolic activation with S9 mix. The cells treated for 6 h were further incubated for 18 h after changing to normal media. After incubation, the cells were collected in 7.5 mM KCl after treatment with 0.2 μg/mL colcemid, and stained with 4% giemsa solution. The number of cells with chromosome aberration per 100 cells was determined from 2 slide glasses.

For the in vivo micronucleus test, 8-week old male ICR mice (Koatech, Korea) were used. Following determination of treatment doses and duration, 4 groups of animals (*n* = 5/group) were orally administered with either vehicle or a dose of FCPE (500, 1000 and 2000 mg/kg BW) daily for 4 days, while 2 mg/kg BW mitomycin c was intraperitoneally injected for the positive control group. Animals were euthanized at 24 h from the last administration and bone marrow cells were collected by perfusion of femora with FBS. The cells were smeared on slide glasses and stained with 5% giemsa solution after fixation. Following treatment with 0.004% citric acid, the number of micronuclei-containing polychromatic erythrocytes (MNPCEs) per 1000 PCEs was determined in two samples per mouse. % PCE was calculated from PCE ÷ [PCE + normochromatic erythrocytes (NCE)].

### Statistical analysis

All values are expressed as mean ± SD. Statistical analysis was performed in a SPSS software (IBM) and a *P* value less than 0.05 was considered as statistically significant.

## Results

### Acute toxicity study

FCPE was prepared from mature fruits collected in Jeju Island, Korea and we analyzed the levels of the reference compounds nobiletin, tangeretin and *p*-synephrine to confirm the validity of FCPE as the test substance for toxicity study. When analyzed using HPLC, the initial levels of nobiletin and tangeretin in raw material were respectively 5.34 ± 0.09 mg/g and 4.43 ± 0.09 mg/g, and increased to 8.22 ± 0.04 mg/g and 5.00 ± 0.14 mg/g by the fermentation process, while *p*-synephrine remained unchanged (3.40 ± 0.04 mg/g to 3.40 ± 0.08 mg/g), which were comparable to the previously reported values [13].

**Fig. 1:**
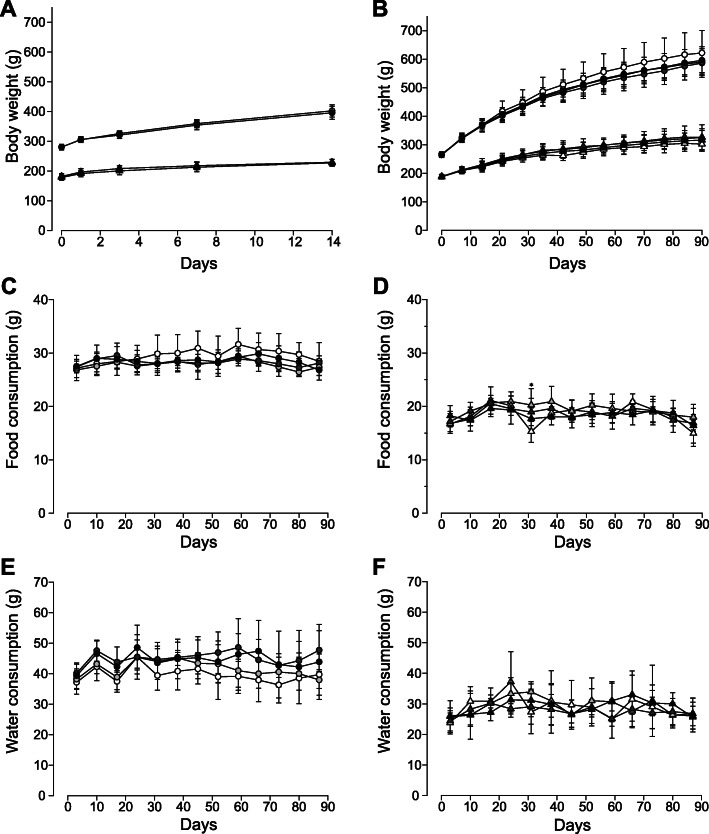
Body weight gain, food and water consumption of SD rats orally administered with fermented *C. sunki* peel extract. Body weight of SD rats was monitored during the toxicity studies of fermented *C. sunki* peel extract (FCPE). **a** Animals (*n* = 5/gender/group, circle for male and triangle for female animals) administered with a single dose of FCPE (open symbols; 0 mg/kg BW and black symbols; 2000 mg/kg BW) in an acute toxicity acquired body weight to a similar degree during the 14-day monitoring period. **b** In the 90-day toxicity study, body weight gain of all SD rats (*n* = 10/gender/group) administered with FCPE was comparable without a remarkable difference for the entire study period. Consumption of food (**c** and **d**) and water (**e** and **f**) was similar across all groups with an exception of higher food intake (*p* < 0.05) in the female 500 mg/kg group in week 5 compared to the vehicle control. Male; circle, female; triangle, 0 mg/kg BW; open symbols, light gray symbols; 500 mg/kg BW, dark gray symbols; 1000 mg/kg BW and black symbols; 2000 mg/kg BW. *; *p <* 0.05 by one-way ANOVA followed by *post-hoc* Dunnett’s test

For the acute toxicity study, we conducted a limit test as the test substance is expected to be non- or weakly toxic based on its natural source and historical use. Following single oral administration of 2000 mg/10 mL/kg BW FCPE, no lethality nor toxicity-associated clinical signs were observed (data not shown), and body weight gain was similar between the vehicle and FCPE-administered groups during the 2-week observation period, (Fig. 1a). At necropsy, we did not find any pathologically meaningful lesions attributable to the test substance. These findings demonstrated that oral administration of FCPE did not cause acute toxicity in SD rats, indicating that the LD50 of FCPE is > 2000 mg/kg BW.

### 90-day repeated oral toxicity study

Based on the results from our acute toxicity study, we performed a 90-day repeated oral toxicity study. SD rats were daily administered with one of 0, 500, 1000 and 2000 mg/kg BW FCPE and examined daily for clinical signs with weekly measurement of body weight. During the entire test period, no animals were found with mortality, clinical signs and behavioral abnormality in relation to FCPE (data not shown). Body weight normally increased in all animals regardless of administered substances and doses (Fig. 1b). Consistently, food and water consumption (Fig. 1c-f) was similar across all groups with an exception of higher food intake (*p* <0.05) in the female 500 mg/kg group in week 5 compared to the vehicle control. Ophthalmological examination (data now shown) and urinalysis (Supplementary Table 1) carried out in the last week of FCPE administration did not find any FCPE-linked toxic effects in the treated groups when compared to their respective vehicle controls.

At the end of 90 days, blood was collected from all animals and analyzed for blood cell counts and coagulation ability (Table 1). Compared to the vehicle control group, male 1000 mg/kg group showed a 5.5% decrease (*p* < 0.05) in lymphocytes, while neutrophils were significantly increased by 35.1% (*p <* 0.05). % Reticulocyes in the male 500 mg/kg group was decreased by 18.5% (*p* < 0.05), but in female group, there was a 25.0% elevation (*p <* 0.05). These changes were considered as incidental and therefore unrelated to the test substance as they were isolated cases without dose-dependency, and there were no other noticeable changes observed in the other FCPE-treated groups. When coagulation ability was assessed, all groups showed comparable levels of the time until formation of coagulation, indicating normal function of the coagulation system in the FCPE-administered groups. Among serum biochemical parameters (Table 2), the levels of glucose in the male 500 mg/kg group showed an increase by 16.4% (*p* < 0.05) compared to the vehicle control without similar change detected in higher dose groups. Besides this, no other parameters showed a statistically meaningful change.

**Table 1:** Hematological parameters of SD rats orally administered with fermented *Citrus Sunki* peel extract for 90 days

	Dose of fermented *Citrus sunk*i peel extract (mg/kg)			
	0	500	1000	2000
*Male (n = 10/group)*				
WBC (10^3^/mm^3^)	9.4 ± 2.4	8.7 ± 3.8	8.4 ± 2.4	8.4 ± 1.0
RBC (10^6^/mm^3^)	8.1 ± 0.4	7.8 ± 0.4	8.2 ± 0.3	7.8 ± 0.5
HGB (g/dl)	14.4 ± 0.7	13.9 ± 0.7	14.6 ± 0.6	14.1 ± 0.6
HCT (%)	39.1 ± 2.0	37.6 ± 2.0	39.3 ± 1.9	35.3 ± 7.6
PLT (10^3^/mm^3^)	789.0 ± 84.8	768.8 ± 71.4	800.2 ± 83.3	724.8 ± 57.3
MCV (fl)	48.3 ± 2.6	48.3 ± 1.5	47.8 ± 1.2	48.8 ± 2.5
MCH (pg)	17.8 ± 0.8	17.9 ± 0.6	17.8 ± 0.4	18.1 ± 0.9
MCHC (g/dl)	36.9 ± 0.7	37.1 ± 0.6	37.2 ± 0.9	37.0 ± 0.6
Neutrophils (%)	11.1 ± 3.1	11.1 ± 3.4	15.0 ± 4.1*	12.0 ± 2.4
Eosinophils (%)	0.30 ± 0.22	0.21 ± 0.17	0.2 ± 0.09	0.15 ± 0.11
Basophils (%)	0.57 ± 0.14	0.50 ± 0.15	0.56 ± 0.18	0.54 ± 0.16
Lymphocytes (%)	83.1 ± 3.7	83.2 ± 4.0	78.5 ± 4.5*	82.1 ± 3.3
Monocytes (%)	3.7 ± 0.6	3.8 ± 0.7	4.5 ± 0.9	4.0 ± 1.0
Reticulocytes (%)	2.7 ± 0.4	2.2 ± 0.2*	2.9 ± 0.6	2.4 ± 0.3
PT (sec)	16.0 ± 0.7	15.7 ± 0.5	15.7 ± 0.7	15.7 ± 0.8
aPTT (sec)	43.0 ± 7.6	44.1 ± 2.0	42.7 ± 2.6	44.0 ± 4.3
*Female (n = 10/group)*				
WBC (10^3^/mm^3^)	7.1 ± 1.6	6.7 ± 1.7	8.0 ± 2.0	7.8 ± 3.1
RBC (10^6^/mm^3^)	7.2 ± 0.2	7.2 ± 0.4	7.1 ± 0.4	7.0 ± 0.3
HGB (g/dl)	13.6 ± 0.5	13.3 ± 0.3	13.3 ± 0.7	13.3 ± 0.4
HCT (%)	36.9 ± 1.1	36.2 ± 1.2	36.2 ± 2.2	36.1 ± 1.2
PLT (10^3^/mm^3^)	700.0 ± 93.5	705.2 ± 41.3	654.3 ± 58.8	710.8 ± 48.6
MCV (fl)	51.5 ± 0.9	50.2 ± 1.9	50.9 ± 2.0	51.3 ± 2.4
MCH (pg)	19.0 ± 0.7	18.5 ± 0.7	18.7 ± 0.6	18.9 ± 0.7
MCHC (g/dl)	36.9 ± 1.0	36.9 ± 0.9	36.8 ± 1.0	36.8 ± 0.7
Neutrophils (%)	7.7 ± 1.4	7.4 ± 1.5	7.4 ± 2.0	8.6 ± 2.9
Eosinophils (%)	0.12 ± 0.09	0.22 ± 0.18	0.17 ± 0.12	0.22 ± 0.19
Basophils (%)	0.41 ± 0.10	0.47 ± 0.15	0.44 ± 0.13	0.42 ± 0.08
Lymphocytes (%)	87.2 ± 2.0	87.4 ± 2.0	87.4 ± 2.5	86.1 ± 3.7
Monocytes (%)	3.3 ± 0.5	3.4 ± 0.5	3.5 ± 0.6	3.6 ± 0.6
Reticulocytes (%)	2.0 ± 0.3	2.5 ± 0.5*	2.5 ± 0.4	2.2 ± 0.4
PT (sec)	15.9 ± 0.8	16.3 ± 0.5	15.9 ± 0.6	15.9 ± 0.6
aPTT (sec)	47.6 ± 5.9	49.5 ± 6.4	44.8 ± 8.0	41.6 ± 7.2

*WBC* white blood cells, *RBC* red blood cells, *Hb* hemoglobin, *HCT* hematocrit, *PLT* platelet, *MCV* mean corpuscular volume, *MCH* mean corpuscular hemoglobin, *MCHC* mean corpuscular hemoglobin concentration, *PT* partial thromboplastin time and *aPTT* activated partial thromboplastin time

^*^; *p* < 0.05 by one-way ANOVA followed by *post-hoc* Dunnet’s test

**Table 2:** Serum biochemical parameters of SD rats orally administered with fermented *Citrus sunki* peel extract for 90 days

	Dose of fermented *Citrus sunk*i peel extract (mg/kg)			
	0	500	1000	2000
*Male (n = 10/group)*				
BUN (mg/dL)	14.4 ± 1.5	13.5 ± 1.5	13.7 ± 1.4	14.1 ± 2.0
TC (mg/dL)	72.4 ± 8.6	69.0 ± 13.7	61.9 ± 8.0	61.9 ± 13.1
TP (g/dL)	6.0 ± 0.4	5.7 ± 0.3	5.8 ± 0.3	6.0 ± 0.2
Albumin (g/dL)	2.4 ± 0.1	2.3 ± 0.1	2.5 ± 0.1	2.4 ± 0.2
TB (mg/dL)	0.00 ± 0.00	0.02 ± 0.04	0.00 ± 0.00	0.00 ± 0.00
ALP (IU/L)	228.9 ± 43.7	208.3 ± 49.5	225.8 ± 61.4	205.7 ± 33.3
AST (IU/L)	112.5 ± 20.7	97.8 ± 17.7	105.7 ± 22.6	92.9 ± 28.6
ALT (IU/L)	34.5 ± 7.6	31.7 ± 2.5	33.8 ± 3.1	31.5 ± 7.7
Creatinine (mg/dL)	0.56 ± 0.04	0.52 ± 0.05	0.51 ± 0.11	0.58 ± 0.06
TG (mg/dL)	59.8 ± 13.6	76.9 ± 26.7	85.1 ± 33.5	71.4 ± 29.8
Glucose (mg/L)	153.1 ± 11.7	178.2 ± 25.7*	154.3 ± 11.9	169.1 ± 25.4
Potassium (mmol/L)	4.9 ± 0.2	4.8 ± 0.3	4.6 ± 0.2	4.8 ± 0.3
Chlorine (mmol/L)	101.4 ± 2.9	102.3 ± 2.6	102.4 ± 1.5	104.2 ± 2.1
Calcium (mg/dL)	9.8 ± 0.5	9.6 ± 0.4	9.5 ± 0.3	10.0 ± 0.3
Phosphorus (mg/dL)	7.0 ± 0.4	6.7 ± 0.5	6.6 ± 0.5	7.1 ± 0.7
Sodium (mmol/L)	138.0 ± 3.1	137.4 ± 2.9	138.5 ± 3.7	140.6 ± 1.8
*Female (n = 10/group)*				
BUN (mg/dL)	13.2 ± 2.3	15.0 ± 1.9	12.7 ± 1.2	13.6 ± 2.1
TC (mg/dL)	76.2 ± 8.0	79.6 ± 15.2	72.7 ± 18.5	71.6 ± 18.6
TP (g/dL)	6.3 ± 0.3	6.3 ± 0.4	6.5 ± 0.5	6.4 ± 0.4
Albumin (g/dL)	2.8 ± 0.2	2.9 ± 0.2	2.9 ± 0.3	2.9 ± 0.3
TB (mg/dL)	0.04 ± 0.05	0.02 ± 0.04	0.01 ± 0.06	0.00 ± 0.00
ALP (IU/L)	112.9 ± 20.6	117.4 ± 36.9	107.1 ± 26.1	101.4 ± 23.7
AST (IU/L)	103.0 ± 23.7	104.1 ± 48.1	90.2 ± 19.6	84.7 ± 14.8
ALT (IU/L)	49.5 ± 10.6	43.0 ± 16.1	40.5 ± 16.4	35.5 ± 5.2
Creatinine (mg/dL)	0.71 ± 0.10	0.62 ± 0.10	0.60 ± 0.22	0.63 ± 0.05
TG (mg/dL)	50.2 ± 38.7	75.4 ± 40.0	75.0 ± 107.8	50.1 ± 35.9
Glucose (mg/L)	203.7 ± 22.1	216.4 ± 17.4	212.2 ± 15.5	201.4 ± 24.1
Potassium (mmol/L)	4.3 ± 0.2	4.5 ± 0.161	4.4 ± 0.1	4.5 ± 0.2
Chlorine (mmol/L)	106.0 ± 2.3	105.2 ± 2.1	105.2 ± 2.4	104.5 ± 2.2
Calcium (mg/dL)	10.5 ± 0.3	10.6 ± 0.2	10.6 ± 0.5	10.5 ± 0.5
Phosphorus (mg/dL)	6.2 ± 0.5	6.3 ± 0.6	6.4 ± 0.6	6.5 ± 0.6
Sodium (mmol/L)	140.9 ± 1.9	140.5 ± 1.3	140.0 ± 1.7	141.3 ± 1.0

*BUN* blood urea nitrogen, *TC* total cholesterol, *TP* total protein, *TB* total bilirubin, *ALP* alkaline phosphatase, *AST* aspartate aminotransferase, *ALT* alanine aminotransferase, and *TG* triglycerides

*; *p* < 0.05 by one-way ANOVA followed by *post-hoc* Dunnett's test

When the organs were excised and weighed at necropsy (Table 3), a significant change (*p* < 0.05) in absolute weight compared to the vehicle group was noted in the left testis (10.0% decrease), the heart (9.5% decrease) and the lung (8.2% decrease) in the male 500 mg/kg group, and in the heart (12.8% decrease), the lung (8.8% decrease) and, the pituitary grand (13.3% decrease) in the male 1000 mg/kg group. In female groups, the left kidney of the highest dose group was markedly increased by 10.1% (*p <* 0.05). On the contrary, the relative weight of all the organs examined were found to be comparable. Gross examination carried out on all organs revealed that a male animal from the 1000 mg/kg group had a 3 mm nodule in the liver and another had a duodenal diverticulum (5 × 7 mm). In female groups, red discolorization was observed in the left thymus and the left cranial lobe of the lung (500 mg/kg group), and the right ovary (1000 mg/kg group). These are likely sporadic legions as only single cases were detected without evident dose dependency and corresponding histopathological changes (Supplementary Table 2). The other histopathological lesions identified in microscopic examination were observed at similar frequencies between the vehicle control and 2000 mg/kg FCPE administered groups and therefore considered as spontaneous or incidental (Supplementary Table 2).

**Table 3:** Absolute and relative organ weight of SD rats orally administered with fermented *Citrus sunki* peel extract for 90 days

		Dose of fermented *Citrus sunk*i peel extract (mg/kg)			
		0	500	1000	2000
*Male (n = 10/group)*					
Liver	(g)	17.1 ± 2.5	15.6 ± 1.5	15.3 ± 1.4	16.1 ± 2.1
	(g%)	2.83 ± 0.12	2.73 ± 0.18	2.737 ± 0.14	2.81 ± 0.18
Spleen	(g)	0.94 ± 0.16	0.84 ± 0.14	0.81 ± 0.09	0.87 ± 0.13
	(g%)	0.16 ± 0.02	0.15 ± 0.02	0.15 ± 0.02	0.15 ± 0.02
Kidney (R)	(g)	2.02 ± 0.19	1.83 ± 0.18	1.85 ± 0.17	1.90 ± 0.14
	(g%)	0.34 ± 0.02	0.32 ± 0.02	0.33 ± 0.03	0.33 ± 0.03
Kidney (L)	(g)	1.99 ± 0.26	1.79 ± 0.21	1.82 ± 0.16	1.92 ± 0.18
	(g%)	0.33 ± 0.02	0.31 ± 0.02	0.38 ± 0.03	0.34 ± 0.02
Adrenal gl. (R)	(g)	0.031 ± 0.004	0.028 ± 0.003	0.029 ± 0.006	0.031 ± 0.006
	(g%)	0.005 ± 0.001	0.005 ± 0.001	0.005 ± 0.001	0.005 ± 0.001
Adrenal gl. (L)	(g)	0.034 ± 0.006	0.029 ± 0.003	0.031 ± 0.005	0.032 ± 0.005
	(g%)	0.006 ± 0.001	0.005 ± 0.001	0.006 ± 0.001	0.006 ± 0.001
Testis (R)	(g)	1.78 ± 0.08	1.62 ± 0.16	1.70 ± 0.18	1.82 ± 0.16
	(g%)	0.30 ± 0.03	0.29 ± 0.03	0.31 ± 0.05	0.32 ± 0.05
Testis (L)	(g)	1.80 ± 0.09	1.62 ± 0.14*	1.73 ± 0.20	1.82 ± 0.16
	(g%)	0.30 ± 0.03	0.29 ± 0.03	0.31 ± 0.06	0.32 ± 0.05
Thymus	(g)	0.28 ± 0.05	0.30 ± 0.10	0.28 ± 0.06	0.24 ± 0.06
	(g%)	0.047 ± 0.009	0.054 ± 0.018	0.051 ± 0.012	0.041 ± 0.009
Heart	(g)	1.79 ± 0.14	1.62 ± 0.13*	1.56 ± 0.15*	1.69 ± 0.16
	(g%)	0.30 ± 0.03	0.29 ± 0.02	0.28 ± 0.01	0.30 ± 0.02
Lung	(g)	1.70 ± 0.20	1.56 ± 0.05*	1.55 ± 0.09*	1.61 ± 0.09
	(g%)	0.28 ± 0.02	0.28 ± 0.02	0.28 ± 0.02	0.28 ± 0.03
Brain	(g)	2.22 ± 0.14	2.14 ± 0.11	2.11 ± 0.09	2.16 ± 0.14
	(g%)	0.37 ± 0.05	0.38 ± 0.03	0.38 ± 0.04	0.38 ± 0.03
Pituitary gl.	(g)	0.015 ± 0.002	0.015 ± 0.002	0.013 ± 0.001*	0.014 ± 0.001
	(g%)	0.002 ± 0.000	0.003 ± 0.000	0.002 ± 0.000	0.002 ± 0.000
*Female (n = 10/group)*					
Liver	(g)	8.2 ± 1.1	9.1 ± 0.9	8.6 ± 1.9	9.0 ± 1.7
	(g%)	2.74 ± 0.26	2.83 ± 0.14	2.63 ± 0.33	2.88 ± 0.25
Spleen	(g)	0.55 ± 0.05	0.57 ± 0.08	0.57 ± 0.07	0.55 ± 0.10
	(g%)	0.18 ± 0.02	0.18 ± 0.02	0.18 ± 0.03	0.18 ± 0.02
Kidney (R)	(g)	0.91 ± 0.07	0.97 ± 0.06	0.98 ± 0.13	1.00 ± 0.09
	(g%)	0.30 ± 0.02	0.30 ± 0.02	0.30 ± 0.02	0.32 ± 0.03
Kidney (L)	(g)	0.89 ± 0.09	0.97 ± 0.04	0.98 ± 0.12	0.98 ± 0.07*
	(g%)	0.30 ± 0.02	0.30 ± 0.02	0.30 ± 0.02	0.32 ± 0.03
Adrenal gl. (R)	(g)	0.033 ± 0.003	0.037 ± 0.004	0.034 ± 0.008	0.034 ± 0.005
	(g%)	0.011 ± 0.001	0.011 ± 0.001	0.010 ± 0.002	0.011 ± 0.001
Adrenal gl. (L)	(g)	0.037 ± 0.005	0.037 ± 0.004	0.036 ± 0.009	0.037 ± 0.006
	(g%)	0.012 ± 0.002	0.011 ± 0.001	0.011 ± 0.003	0.012 ± 0.001
Ovary (R)	(g)	0.060 ± 0.007	0.113 ± 0.158	0.059 ± 0.010	0.061 ± 0.015
	(g%)	0.020 ± 0.003	0.038 ± 0.058	0.019 ± 0.004	0.020 ± 0.005
Ovary (L)	(g)	0.058 ± 0.009	0.056 ± 0.010	0.058 ± 0.013	0.058 ± 0.009
	(g%)	0.019 ± 0.003	0.018 ± 0.004	0.018 ± 0.005	0.019 ± 0.004
Thymus	(g)	0.25 ± 0.06	0.28 ± 0.05	0.26 ± 0.07	0.29 ± 0.10
	(g%)	0.084 ± 0.018	0.088 ± 0.016	0.083 ± 0.023	0.092 ± 0.023
Heart	(g)	0.94 ± 0.09	1.05 ± 0.10	0.99 ± 0.10	0.97 ± 0.10
	(g%)	0.32 ± 0.03	0.33 ± 0.03	0.31 ± 0.03	0.31 ± 0.02
Lung	(g)	1.23 ± 0.06	1.25 ± 0.16	1.26 ± 0.20	1.22 ± 0.08
	(g%)	0.41 ± 0.03	0.39 ± 0.05	0.40 ± 0.08	0.40 ± 0.05
Brain	(g)	1.92 ± 0.07	1.95 ± 0.06	1.95 ± 0.09	1.90 ± 0.06
	(g%)	0.64 ± 0.04	0.61 ± 0.05	0.62 ± 0.08	0.62 ± 0.08
Pituitary gl.	(g)	0.016 ± 0.003	0.017 ± 0.002	0.018 ± 0.003	0.019 ± 0.005
	(g%)	0.005 ± 0.001	0.005 ± 0.001	0.006 ± 0.001	0.006 ± 0.001

^*^; *p* < 0.05 by one-way ANOVA followed by *post-hoc* Dunnett’s test

Collectively, the combined results of our acute and 90-day repeated toxicity study demonstrated that FCPE was safe for oral consumption up to 2000 mg/kg BW in SD rats, suggesting that its no-observed-adverse-effect-level (NOAEL) is higher than 2000 mg/kg BW.

### Bacterial reverse mutation test

**Fig. 2:**
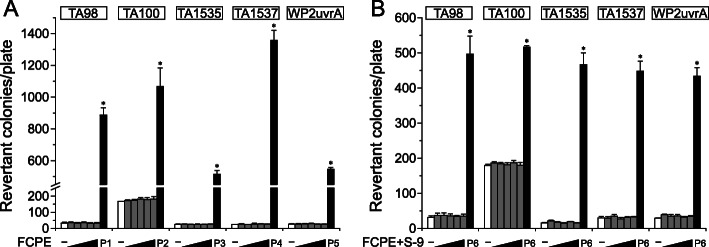
Absence of reverse mutation capacity in fermented *C. sunki* peel extract. Five bacterial tester strains (4 histidine auxotrophs *S. typhimurium* TA98, TA100, TA1535 and TA1537 and 1 tryptophan auxotroph *E. coli* WP2(*uvrA*)) were employed to assess reverse mutation capacity of fermented *C. sunki* peel extract (FCPE). Treatment of the tester strains with increasing doses of FCPE (gray bars; 312.5, 625, 1250, 2500 and 5000 μg/plate) resulted in the similar numbers of revertant colonies to the vehicle control (white bars) in the absence (**a**) and presence (**b**) of the S-9 factor mediated metabolic activation, while significant increase in the colony number by positive controls (black bars). P1; 10 μg/plate 2-nitrofluroene, P2; 5 μg/plate sodium azide, P3; 0.5 μg/plate sodium azide, P4; 80 μg/plate 9-aminoacridine, P5; 0.5 μg/plate mitomycin C, and P6; 2-aminoanthracene. *; *p* < 0.05 by one-way ANOVA followed by Tukey’s HSD multiple comparison test

Next, we examined the genotoxicity of FCPE by assessing bacterial mutation reversion, chromosome aberration and in vivo micronucleus formation. FCPE was not cytotoxic to TA100 tester strain up to 5000 μg/plate as similar numbers of colonies were observed regardless of the presence of S-9 factor, while positive controls induced a marked increase of revertant colonies (Supplementary Table 3). To test reversion of mutation, we pre-treated up to 5000 μg/plate FCPE on 5 tester strains including 4 histidine-requiring *S. typhimurium* TA98, TA100, TA1535, TA1537 and a tryptophan-requiring *E. coli* WP2(*uvrA*) and counted the number of colonies formed on histidine-deficient or tryptophan-deficient agar plates as the indicator of mutation reversion (Fig. 2). Treatment with FCPE resulted in similar numbers of colonies to the vehicle control in all the strains tested in the study, while the positive controls significantly increased their number. Moreover, metabolic activation of FCPE using S-9 mix also did not effectively increase the colony number, collectively indicating inability of FCPE to induce mutations in bacterial tester strains.

### In vitro chromosome aberration test

**Fig. 3:**
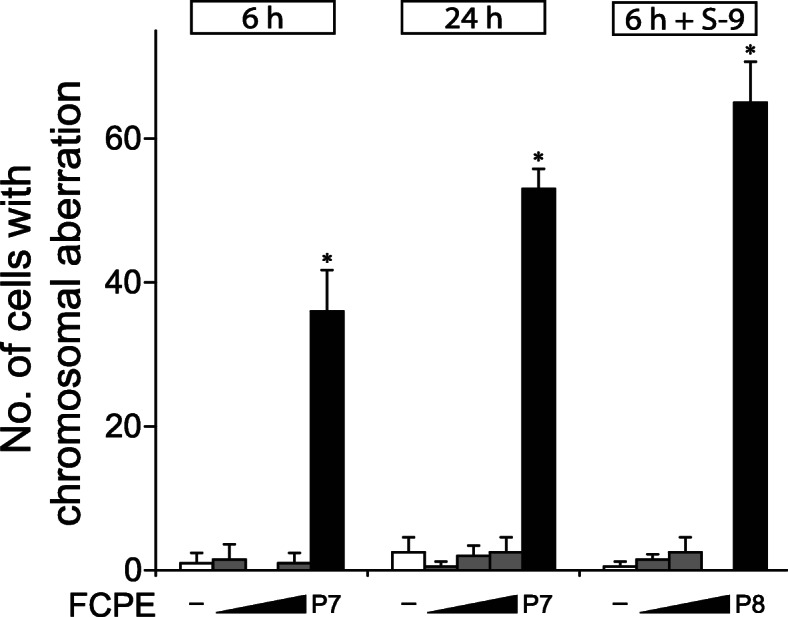
Lack of chromosome aberration by fermented *C. sunki* peel extract. Chromosomal aberration test was performed using Chinese Hamster Lung cells. Cells were treated with 1250, 2500 and 5000 μg/mL of fermented *C. sunki* peel extract (FCPE) for the indicated time with or without metabolic activation using the S-9 factor, and examined to identify cells containing aberrant chromosomes. While the positive controls (P7; mitomycin C and P8; cyclophosphamide, black bars) significantly increased the number of cells with chromosome aberration, FCPE (gray bars) did not show a noticeable change compared to the control (white bars) in all tested conditions. *; *p* < 0.05 when compared to the control by Fisher’s exact test

Chromosome aberration was measured in 200 CHL cells in metaphase after treatment with FCPE. Notably, FCPE did not affect cell viability at up to 5000 μg/mL (Supplementary Table 4). Compared to the significantly increased number of cells with chromosome aberration in the positive control groups (Fig. 3), all doses of FCPE used here resulted in a similar number of chromosomally aberrant cells to the vehicle control irrespective of S-9 mediated metabolic activation.

### In vivo micronucleus formation test

**Fig. 4:**
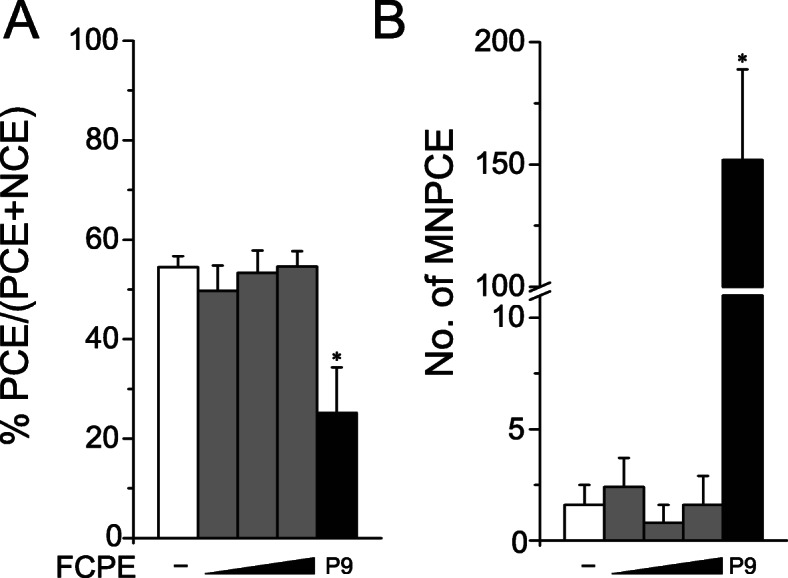
Normal levels of micronucleus formation by fermented *C. sunki* peel extract. Bone marrow cells collected from male ICR mice (*n* = 5/group) orally treated with 500, 1000 or 2000 mg/kg BW fermented *C. sunki* peel extract (FCPE) were examined to determine (**a**) % polychromatic erythrocytes (PCE) and (**b**) the number of micronuclei-containing polychromatic erythrocytes (MNPCE) per 1000 polychromatic erythrocytes (PCE). FCPE (gray bars) showed comparable levels of % PCE as well as MNPCE counts to the vehicle control (white bars), while mitomycin C (P9, black bars) induced a significant decrease in % PCE with a marked increase of MNPCE. PCE; polychromatic erythrocytes, NCE; normochromatic erythrocytes, MNPCE; polychromatic erythrocytes with micronuclei. *; *p <* 0.05 and **; *p* < 0.01 by Kruskal-Wallis one-way ANOVA followed by *post-hoc* Tukey’s HSD multiple comparison test

The results of above assays suggested that FCPE lack in vitro genotoxicity. To confirm these findings in an in vivo system, we administered FCPE in ICR mice and examined the number of micronuclei-containing polychromatic erythrocytes (MNPCE) as a readout of genotoxicity (Fig. 4). Oral administration of up to 2000 mg/kg BW FCPE for 4 days did not induce any change in either % PCE (Fig. 4a) or the number of MNPCE (Fig. 4b) at any doses compared to the vehicle control, while treatment with mitomycin C resulted in a marked increase. Collectively, these findings demonstrated that FCPE had no genotoxic potential in our test system.

## Discussion

*C. sunki* peel has been a popular ingredient due to its empirically tested therapeutic effects in traditional folk medicine. Despite the long history of its use, toxicity information of *C. sunki* peel has not been available partly due to small-scaled usage along with a low rate of side effects. However, a growing body of evidence on its pharmacological effects from recent studies led to development of highly concentrated products and increased consumption, posing a higher risk of over-dosing. In this study, we systematically assessed the safety of water extract of fermented *C. sunki* peel by carrying out a series of systemic toxicity studies in combination with in vitro and in vivo genotoxicity assays. Our results on FCPE in this study were generally in line with the previous studies on other citrus species, but also there were different findings.

Among a handful of citrus species whose toxicity has been preclinically evaluated, *C. aurantium L.* (bitter orange) extract has been most extensively studied [29]; > 5000 mg/kg for LD50 [30], 1000 mg/kg for NOAEL [31] and no developmental toxicity at 100 mg/kg [32]. A recent study on *C. unshiu* Marcow. peel water extract also reported low toxicity with > 4000 mg/kg for LD50 and NOAEL [33]. Our findings of 2000 mg/kg FCPE for LD50 and NOAEL are closely consistent with these reports, suggesting the safety of FCPE for human consumption and recapitulating the general safety of citrus species peel by adding another layer of evidence.

Among the polymethoxy flavonoids in *C. sunki* peel, tangeretin is one of the most abundant components and has been associated with in vivo toxicity in mice; Although LD50 was > 3000 mg/kg BW, repeated administration of 50 mg/kg caused reduction of relative liver weight with hepatocellular alteration, and increased triglycerides and total cholesterol of female groups in a 28-day oral toxicity study [34]. Also, tangeretin was reported to be a mild immunotoxicant as demonstrated in a concurrent reduction of T cell-derived interleukins and Th17 cell population by oral administration of 50 mg/kg tangeretin [35] and reduction of circulating lymphocytes by 0.22 mg/day [36]. In our 90-day toxicity study, we did not observe such pathological changes in the SD rats fed with 2000 mg/kg FCPE (equivalent to 6.8 mg/kg/day tangeretin) except for mild reduction of circulating lymphocytes in the male 1000 mg/kg group. It is tempting to propose that the observed discrepancy may be caused due to the different species we used in our study (i.e., SD rats instead of mice) or the low levels of tangeretin contained in FCPE compared to the doses employed in the mice studies, but the responsible mechanism may need to be clarified in further investigation.

Genotoxicity of citrus species have been controversial with contradicting evidence reported from several studies. While no genotoxicity was detected in three studies on *C. aurantium L.* (bitter orange) extract [30], *C. unshiu* Marcow. peel water extract [33] and polymethoxy flavones extracted from citrus peel oil [18], Franke et al. showed that juice prepared from *C. sinensis* Linn. had a mild degree of mutagenicity in the Ames test [37] and the comet assay [38]. Our results on FCPE demonstrated no mutagenicity and clastogenicity, supporting lack of genotoxic potential in citrus peel. The difference observed among the studies may be explained by distinct composition of citrus components derived from usage of different species, part of citrus and extract methods. As quercetin and kaempferol have been detected as mutagenic in a mammalian cell line [39], quantification of these flavonols may be of great interest to understand their contribution to the differential responses of citrus species in genotoxicity assays. Although the responsible compounds for mutagenicity remain to be identified, currently accumulated evidence including our results collectively suggest that citrus peel have no genotoxicity.

In this study, we systematically assessed general and genetic toxicity of fermented *C. sunki* peel extract using in vivo and in vitro models, providing the toxicity information to be used for safe consumption in humans and adding another layer of evidence for the general safety of citrus species. As a health supplement, the oral dose range of commercial citrus peel extract is generally recommended to be 40 ~ 100 mg/kg/day in human with the usual duration of administration from days up to a month. Therefore, our findings suggest that FCPE may be consumed without harmful effects in human following the current guideline.

## Conclusions

FCPE, under the test conditions we employed in the study, demonstrated no systemic toxicity in SD rats, establishing > 2000 mg/kg BW for LD50 as well as NOAEL, and absence of mutagenicity and clastogenicity at 5000 μg/plate for in vitro assays and 2000 mg/kg BW for in vivo micronucleus test.

## Supplementary information

**Additional file 1 Supplementary Table 1**. Urinalysis of SD rats orally treated with fermented *C. sunki* peel extract for 90 days. **Supplementary Table 2.** Pathological findings in major organs from SD rats orally treated with fermented *C. sunki* peel extract for 90 days. **Supplementary Table 2.** Pathological findings in major organs from SD rats orally treated with fermented *C. sunki* peel extract for 90 days (continued from the previous page). **Supplementary Table 3.** Cytotoxicity of fermented *C. sunki* peel extract on *S. tiphimurium* TA100. **Supplementary Table 4.** MTT assay results of fermented *C. sunki* peel extract on Chinese hamster lung cells.

## Data Availability

The datasets used and/or analyzed during the current study have been archived in Department of Experimental Animal Research, Biomedical Research Institute, Seoul National University Hospital, and are available from the corresponding author on reasonable request.

## Abbreviations

ALP: Alkaline phosphatase
ALT: Alanine aminotransferase
aPTT: Activated partial thromboplastin time
AST: Aspartate aminotransferase
BUN: Blood urea nitrogen
CHL: Chinese Hamster Lung
EDTA: Ethylenediaminetetraacetic acid
FBS: Fetal bovine serum
FCPE: Fermented *C. sunki* peel extract
Hb: Hemoglobin
HCT: Hematocrit
KFDA: Korea Food Drug Administration
LD50: Lethal dose 50%
MCH: Mean corpuscular hemoglobin
MCHC: Mean corpuscular hemoglobin concentration
MCV: Mean corpuscular volume
MNPCE: Micronuclei-containing polychromatic erythrocyte
NCE: Normochromatic erythrocyte
NOAEL: No-observed-adverse-effect-level
OECD: Organization for Economic Co-operation and Development
PCE: Polychromatic erythrocyte
PLT: Platelet
PT: Partial thromboplastin time
RBC: Red blood cell
TB: Total bilirubin
TC: Total cholesterol
TG: Triglyceride
TP: Total protein
WBC: White blood cell
