# Supplementary material for: In vivo and in vitro safety evaluation of fermented Citrus sunki peel extract: acute and 90-day repeated oral toxicity studies with genotoxicity assessment
Source: BMC Complement Med Ther. 2020 Oct 6;20:297. doi: 10.1186/s12906-020-03079-z (PMC7542383; doi:10.1186/s12906-020-03079-z)
Supplement: Supplementary file 1 — Additional file 1 Supplementary Table 1. Urinalysis of SD rats orally treated with fermented C. sunki peel extract for 90 days. Supplementary Table 2. Pathological findings in major organs from SD rats orally treated with fermented C. sunki peel extract for 90 days. Supplementary Table 2. Pathological findings in major organs from SD rats orally treated with fermented C. sunki peel extract for 90 days (continued from the previous page). Supplementary Table 3. Cytotoxicity of fermented C. sunki peel extract on S. tiphimurium TA100. Supplementary Table 4. MTT assay results of fermented C. sunki peel extract on Chinese hamster lung cells. [file 12906_2020_3079_MOESM1_ESM.docx]

| **Supplementary Table 1**. Urinalysis of SD rats orally treated with fermented *C. sunki* peel extract for 90 days | | | | | | | | | | | | | | | | | |
| --- | --- | --- | --- | --- | --- | --- | --- | --- | --- | --- | --- | --- | --- | --- | --- | --- | --- |
| Parameter | Value |  | Dose of fermented *C. sunki* peel extract (mg/kg) | | | | | | | | | | | | | | |
|  |  |  |  |  | Male (n=5/group) | | | |  |  |  |  | Female (n=5/group) | | |  |  |
|  |  |  | 0 |  | 500 |  | 1000 |  | 2000 |  | 0 |  | 500 |  | 1000 |  | 2000 |
| Specific gravity |  |  | 1.022 ±0.006 |  | 1.032 ±0.007 |  | 1.013 ±0.004 |  | 1.030 ±0.016 |  | 1.023 ±0.008 |  | 1.018 ±0.010 |  | 1.022 ±0.008 |  | 1.030 ±0.013 |
| pH | 6.5 |  | 0 |  | 0 |  | 0 |  | 0 |  | 0 |  | 0 |  | 0 |  | 2 |
|  | 7.0 |  | 0 |  | 0 |  | 0 |  | 0 |  | 0 |  | 1 |  | 0 |  | 0 |
|  | 8.0 |  | 0 |  | 1 |  | 0 |  | 2 |  | 3 |  | 1 |  | 2 |  | 0 |
|  | 9.0 |  | 5 |  | 4 |  | 5 |  | 3 |  | 2 |  | 3 |  | 3 |  | 3 |
| Leukocytes | 0 |  | 0 |  | 0 |  | 0 |  | 0 |  | 2 |  | 4 |  | 0 |  | 2 |
| (cells/µL) | 25 |  | 0 |  | 1 |  | 0 |  | 0 |  | 2 |  | 0 |  | 3 |  | 1 |
|  | 100 |  | 3 |  | 1 |  | 5 |  | 3 |  | 1 |  | 1 |  | 2 |  | 1 |
|  | 500 |  | 2 |  | 3 |  | 0 |  | 2 |  | 0 |  | 0 |  | 0 |  | 1 |
| Nitrite | - |  | 3 |  | 2 |  | 2 |  | 0 |  | 1 |  | 2 |  | 0 |  | 1 |
|  | + |  | 2 |  | 3 |  | 3 |  | 5 |  | 4 |  | 3 |  | 5 |  | 4 |
| Protein (mg/dL) | 0 |  | 0 |  | 1 |  | 0 |  | 0 |  | 2 |  | 4 |  | 0 |  | 1 |
|  | 25 |  | 2 |  | 2 |  | 5 |  | 3 |  | 3 |  | 1 |  | 5 |  | 2 |
|  | 75 |  | 3 |  | 2 |  | 0 |  | 0 |  | 0 |  | 0 |  | 0 |  | 2 |
|  | 150 |  | 0 |  | 0 |  | 0 |  | 2 |  | 0 |  | 0 |  | 0 |  | 0 |
| Glucose (mg/dL) | normal |  | 5 |  | 5 |  | 5 |  | 5 |  | 5 |  | 5 |  | 5 |  | 5 |
| Ketone | - |  | 0 |  | 0 |  | 5 |  | 1 |  | 2 |  | 3 |  | 1 |  | 1 |
|  | + |  | 2 |  | 2 |  | 0 |  | 1 |  | 0 |  | 1 |  | 2 |  | 1 |
|  | ++ |  | 3 |  | 3 |  | 0 |  | 1 |  | 3 |  | 1 |  | 2 |  | 2 |
|  | +++ |  | 0 |  | 0 |  | 0 |  | 2 |  | 0 |  | 0 |  | 0 |  | 1 |
| Urobilinogen | normal |  | 5 |  | 5 |  | 5 |  | 4 |  | 4 |  | 5 |  | 4 |  | 4 |
| (mg/dl) | 1 |  | 0 |  | 0 |  | 0 |  | 1 |  | 1 |  | 0 |  | 1 |  | 0 |
|  | 4 |  | 0 |  | 0 |  | 0 |  | 0 |  | 0 |  | 0 |  | 0 |  | 1 |
| Bilirubin | - |  | 5 |  | 5 |  | 5 |  | 3 |  | 5 |  | 5 |  | 4 |  | 5 |
| (neg./+/++/+++) | + |  | 0 |  | 0 |  | 0 |  | 2 |  | 0 |  | 0 |  | 1 |  | 0 |
| Hemoglobin | 0 |  | 1 |  | 1 |  | 0 |  | 0 |  | 4 |  | 5 |  | 5 |  | 4 |
| (RBC/µL) | 10 |  | 1 |  | 2 |  | 1 |  | 1 |  | 1 |  | 0 |  | 0 |  | 1 |
|  | 25 |  | 3 |  | 2 |  | 4 |  | 2 |  | 0 |  | 0 |  | 0 |  | 0 |
|  | 50 |  | 0 |  | 0 |  | 0 |  | 2 |  | 0 |  | 0 |  | 0 |  | 0 |

| **Supplementary Table 2.** Pathological findings in major organs from SD rats orally treated with fermented *C.*  *sunki* peel extract for 90 days | | | | | | | | | | | | | | | | | | | |  |
| --- | --- | --- | --- | --- | --- | --- | --- | --- | --- | --- | --- | --- | --- | --- | --- | --- | --- | --- | --- | --- |
| Organ | Findings | | | |  | Dose of fermented *C. sunk*i peel extract (mg/kg) | | | | | | | | | | | | | | |
|  |  |  |  |  |  | Male  (n=10/group) | | | | | | |  | Female (n=10/group) | | | | | | |
|  |  |  |  |  |  | 0 | | |  | 2000 | | |  | 0 | | |  | 2000 | | |
| *Nervous system* |  | | | |  |  | | |  |  | | |  |  | | |  |  | | |
| Brain | Normal | | | |  | 10/10 | | |  | 10/10 | | |  | 10/10 | | |  | 10/10 | | |
| Spinal cord | Normal | | | |  | 10/10 | | |  | 10/10 | | |  | 10/10 | | |  | 10/10 | | |
| Sciatic nerve | Normal | | | |  | 10/10 | | |  | 10/10 | | |  | 10/10 | | |  | 10/10 | | |
| *Ocular system* |  | | | |  |  | | |  |  | | |  |  | | |  |  | | |
| Eyes | Normal | | | |  | 10/10 | | |  | 9/10 | | |  | 10/10 | | |  | 10/10 | | |
|  | Retinal detachment with organization | | | |  | 0/10 | | |  | 1/10 | | |  | 0/10 | | |  | 0/10 | | |
| Haderian glands | Normal | | | |  | 10/10 | | |  | 10/10 | | |  | 10/10 | | |  | 10/10 | | |
| *Digestive system* |  | | | |  |  | | |  |  | | |  |  | | |  |  | | |
| Tongue/larynx | Normal | | | |  | 10/10 | | |  | 10/10 | | |  | 10/10 | | |  | 10/10 | | |
| Salivary glands | Normal | | | |  | 8/10 | | |  | 10/10 | | |  | 10/10 | | |  | 10/10 | | |
|  | Focal inflammation | | | |  | 2/10 | | |  | 0/10 | | |  | 0/10 | | |  | 0/10 | | |
| Esophagus | Normal | | | |  | 10/10 | | |  | 10/10 | | |  | 10/10 | | |  | 10/10 | | |
| Stomach | Normal | | | |  | 10/10 | | |  | 10/10 | | |  | 10/10 | | |  | 10/10 | | |
| Duodenum | Normal | | | |  | 10/10 | | |  | 10/10 | | |  | 10/10 | | |  | 10/10 | | |
| Jejunum | Normal | | | |  | 10/10 | | |  | 10/10 | | |  | 10/10 | | |  | 10/10 | | |
| Ileum | Normal | | | |  | 10/10 | | |  | 10/10 | | |  | 10/10 | | |  | 10/10 | | |
| Cecum | Normal | | | |  | 10/10 | | |  | 10/10 | | |  | 10/10 | | |  | 10/10 | | |
| Colon | Normal | | | |  | 10/10 | | |  | 10/10 | | |  | 10/10 | | |  | 10/10 | | |
| Rectum | Normal | | | |  | 10/10 | | |  | 10/10 | | |  | 10/10 | | |  | 10/10 | | |
| Liver | Normal | | | |  | 10/10 | | |  | 9/10 | | |  | 10/10 | | |  | 10/10 | | |
|  | Focal perivascular inflammation | | | |  | 0/10 | | |  | 1/10 | | |  | 0/10 | | |  | 0/10 | | |
| Pancreas | Normal | | | |  | 8/10 | | |  | 6/10 | | |  | 9/10 | | |  | 6/10 | | |
|  | Focal chronic inflammation | | | |  | 2/10 | | |  | 4/10 | | |  | 1/10 | | |  | 0/10 | | |
| *Immune system* |  | | | |  |  | | |  |  | | |  |  | | |  |  | | |
| Thymus | Normal | | | |  | 9/9* | | |  | 10/10 | | |  | 10/10 | | |  | 10/10 | | |
| Spleen | Normal | | | |  | 10/10 | | |  | 10/10 | | |  | 10/10 | | |  | 10/10 | | |
| Cervical lymph node | Normal | | | |  | 10/10 | | |  | 10/10 | | |  | 10/10 | | |  | 10/10 | | |
| Mesenteric lymph node | Normal | | | |  | 10/10 | | |  | 10/10 | | |  | 10/10 | | |  | 0/10 | | |
| *Endocrine system* |  | | | |  |  | | |  |  | | |  |  | | |  |  | | |
| Pituitary gland | Normal | | | |  | 10/10 | | |  | 10/10 | | |  | 10/10 | | |  | 10/10 | | |
| Thyroid glands | Normal | | | |  | 10/10 | | |  | 10/10 | | |  | 10/10 | | |  | 10/10 | | |
| Parathyroid glands | Normal | | | |  | 5/5* | | |  | 7/7* | | |  | 6/6* | | |  | 6/6* | | |
| Adrenal glands | Normal | | | |  | 10/10 | | |  | 9/10 | | |  | 10/10 | | |  | 10/10 | | |
|  | Focal interstitial inflammation with calcification | | | |  | 0/10 | | |  | 1/10 | | |  | 0/10 | | |  | 10/10 | | |
| *; Total number of organs prepared and examined is shown. | | | | |  |  | | |  |  | | |  |  | | |  |  | | |
| **Supplementary Table 2.** Pathological findings in major organs from SD rats orally treated with fermented *C.*  *sunki* peel extract for 90 days (continued from the previous page) | | | | | | | | | | | | | | | | | | |  |  |
| Organ | | Findings |  | Dose of fermented *C. sunk*i peel extract (mg/kg) | | | | | | | | | | | | | | |  |  |
|  |  |  |  | Male  (n=10/group) | | | | | | |  | Female (n=10/group) | | | | | | |  |  |
|  |  |  |  | 0 | | |  | 2000 | | |  | 0 | | |  | 2000 | | |  |  |
| Nasal cavity | | Normal |  | 10/10 | | |  | 10/10 | | |  | 10/10 | | |  | 10/10 | | |  |  |
| Treachea | | Normal |  | 10/10 | | |  | 10/10 | | |  | 10/10 | | |  | 10/10 | | |  |  |
| Lung | | Normal |  | 10/10 | | |  | 10/10 | | |  | 10/10 | | |  | 10/10 | | |  |  |
| *Cardiovascular system* | | |  |  | | |  |  | | |  |  | | |  |  | | |  |  |
| Heart | | Normal |  | 2/10 | | |  | 5/10 | | |  | 9/10 | | |  | 10/10 | | |  |  |
|  | | Focal myocarditis |  | 8/10 | | |  | 3/10 | | |  | 1/10 | | |  | 0/10 | | |  |  |
|  | | Focal myocarditis & endocarditis |  | 0/10 | | |  | 1/10 | | |  | 0/10 | | |  | 0/10 | | |  |  |
|  | | Focal endocarditis |  | 0/10 | | |  | 1/10 | | |  | 0/10 | | |  | 0/10 | | |  |  |
| *Urinary sytstem* | |  |  |  | | |  |  | | |  |  | | |  |  | | |  |  |
| Kidneys | | Normal |  | 4/10 | | |  | 7/10 | | |  | 9/10 | | |  | 0/10 | | |  |  |
|  | | Focal interstitial inflammation |  | 5/10 | | |  | 3/10 | | |  | 0/10 | | |  | 1/10 | | |  |  |
|  | | Focal interstitial inflammation with calcification |  | 1/10 | | |  | 0/10 | | |  | 0/10 | | |  | 3/10 | | |  |  |
|  | | Interstitial inflammation with calcification |  | 0/10 | | |  | 0/10 | | |  | 0/10 | | |  | 10/10 | | |  |  |
|  | | Calcification |  | 0/10 | | |  | 0/10 | | |  | 1/10 | | |  | 0/10 | | |  |  |
| Urinary bladder | | Normal |  | 10/10 | | |  | 10/10 | | |  | 10/10 | | |  | 10/10 | | |  |  |
| *Reproductive system* | | |  |  | | |  |  | | |  |  | | |  |  | | |  |  |
| Preputial gland /Clitoral glands | | Normal |  | 8/10 | | |  | 10/10 | | |  | 10/10 | | |  | 9/10 | | |  |  |
|  | | Focal chronic granulomatous inflammation |  | 1/10 | | |  | 0/10 | | |  | 0/10 | | |  | 0/10 | | |  |  |
|  | | Focal chronic inflammation |  | 1/10 | | |  | 0/10 | | |  | 0/10 | | |  | 1/10 | | |  |  |
| Testes | | Normal |  | 10/10 | | |  | 10/10 | | |  | 10/10 | | |  | 10/10 | | |  |  |
| Epididymides | | Normal |  | 9/10 | | |  | 9/10 | | |  | N/A | | |  | N/A | | |  |  |
|  | | Focal chronic inflammation |  | 1/10 | | |  | 0/0 | | |  | N/A | | |  | N/A | | |  |  |
|  | | Focal chronic granulomatous inflammation |  | 0/0 | | |  | 1/10 | | |  | N/A | | |  | N/A | | |  |  |
| Prostate | | Normal |  | 3/10 | | |  | 2/10 | | |  | N/A | | |  | N/A | | |  |  |
|  | | Chronic inflammation |  | 7/10 | | |  | 5/10 | | |  | N/A | | |  | N/A | | |  |  |
|  | | Chronic inflammation with focal abscess |  | 0/0 | | |  | 3/10 | | |  | N/A | | |  | N/A | | |  |  |
| Seminal vesicle | | Normal |  | 10/10 | | |  | 10/10 | | |  | N/A | | |  | N/A | | |  |  |
| Ovaries | | Normal |  | N/A | | |  | N/A | | |  | 10/10 | | |  | 10/10 | | |  |  |
| Uterus | | Normal |  | N/A | | |  | N/A | | |  | 10/10 | | |  | 10/10 | | |  |  |
| Vagina | | Normal |  | N/A | | |  | N/A | | |  | 10/10 | | |  | 10/10 | | |  |  |
| *Musculoskeletal and other systems* | | |  |  | | |  |  | | |  |  | | |  |  | | |  |  |
| Skin/mammary gland | | Normal |  | 10/10 | | |  | 10/10 | | |  | 10/10 | | |  | 10/10 | | |  |  |
| Skeletal muscle | | Normal |  | 10/10 | | |  | 10/10 | | |  | 10/10 | | |  | 10/10 | | |  |  |
| Thoracic vertebra | | Normal |  | 10/10 | | |  | 10/10 | | |  | 10/10 | | |  | 10/10 | | |  |  |
| Femur /bone marrow | | Normal |  | 10/10 | | |  | 10/10 | | |  | 10/10 | | |  | 10/10 | | |  |  |
| *; Total number is reduced due to loss during tissue preparation | | |  |  | | |  |  | | |  |  | | |  |  | | |  |  |

| **Supplementary table 3.** Cytotoxicity of fermented *C. sunki* peel extract on *S. tiphimurium* TA100 | | | |
| --- | --- | --- | --- |
| S-9 mix | Test substance | Concentration  (µg/100 µL/plate) | His+ revertant colonies/plate |
|  |  |  | TA100 |
| S-9 mix (-) | DW | 0.0 | 195 ± 8.6 |
|  | Fermented *C. sunki* peel extract | 5000.0 | 184 ± 16.1 |
|  |  | 2500.0 | 200 ± 12.3 |
|  |  | 1250.0 | 200 ± 9.2 |
|  |  | 625.0 | 198 ± 17.4 |
|  |  | 312.5 | 216 ± 13.3 |
|  | Sodium azide | 5.0 | 1,126 ± 103.4* |
| S-9 mix (+) | DW | 0.0 | 199 ± 11.0 |
|  | Fermented *C. sunki* peel extract | 5000.0 | 197 ± 30.7 |
|  |  | 2500.0 | 212 ± 19.9 |
|  |  | 1250.0 | 233 ± 23.3 |
|  |  | 625.0 | 240 ± 13.0 |
|  |  | 312.5 | 198 ± 24.4 |
|  | 2-aminoanthracene | 2.0 | 956 ± 40.9* |
| *, Significantly different from the control (*p*<0.05) | | | |

| **Supplementary table 4.** MTT assay results of fermented *C. sunki* peel extract on Chinese hamster lung cells | | | |
| --- | --- | --- | --- |
| Test substance | Concentration (µg/mL) | Absorbance at 540nm | Viability (%) |
| DW | 0.0 | 1.070 ± 0.141 | 100.0 |
| Fermented *C. sunki* peel extract | 5,000.0 | 0.641 ± 0.028 | 59.9 |
|  | 2,500.0 | 0.676 ± 0.050 | 63.2 |
|  | 1,250.0 | 0.787 ± 0.061 | 73.6 |
|  | 625.0 | 0.978 ± 0.053 | 91.4 |
|  | 312.5 | 1.033 ± 0.054 | 96.5 |
|  | 156.3 | 1.100 ± 0.101 | 102.8 |
|  | 78.1 | 1.068 ± 0.101 | 99.8 |
|  | 39.1 | 1.006 ± 0.064 | 94.0 |
